# Research of Wood Waste as a Potential Filler for Loose-Fill Building Insulation: Appropriate Selection and Incorporation into Polyurethane Biocomposite Foams

**DOI:** 10.3390/ma13235336

**Published:** 2020-11-25

**Authors:** Nerijus Augaitis, Saulius Vaitkus, Sylwia Członka, Agnė Kairytė

**Affiliations:** 1 Laboratory of Thermal Insulating Materials and Acoustics, Institute of Building Materials, Faculty of Civil Engineering, Vilnius Gediminas Technical University, 10221 Vilnius, Lithuania; nerijus.augaitis@vgtu.lt (N.A.); agne.kairyte@vgtu.lt (A.K.); 2 Institute of Polymer and Dye Technology, Lodz University of Technology, 90-924 Lodz, Poland; sylwia.czlonka@edu.p.lodz.pl

**Keywords:** wood waste, loose-fill insulation, recycling, polyurethane foam, waste-based aggregates, performance properties, circular economy

## Abstract

Currently, the recycling potential of wood waste (WW) is still limited, and in a resource efficiency approach, recycling WW in insulation materials, such as polyurethane (PUR), appears as an appropriate solution. It is known that the quality of WW is the main aspect which influences the stability of the final products. Therefore, the current study analyses different WW-based fillers as possible modifiers for polyurethane biocomposite foams for the application as loose-fill materials in building envelopes. During the study of WW-based fillers, it was determined that the most promising filler is wood scobs (WS) with a thermal conductivity of 0.0496 W/m·K, short-term water absorption by partial immersion—12.5 kg/m^2^, water vapour resistance—0.34 m^2^·h·Pa/mg and water vapour diffusion resistance factor—2.4. In order to evaluate the WS performance as a filler in PUR biocomposite foams, different ratios of PUR binder and WS filler (PURb/WS) were selected. It was found that a 0.40 PURb/WS ratio is insufficient for the appropriate wetting of WS filler while a 0.70 PURb/WS ratio produced PUR biocomposite foams with the most suitable performance: thermal conductivity reduced from 0.0523 to 0.0476 W/m·K, water absorption—from 5.6 to 1.3 kg/m^2^, while the compressive strength increased from 142 to 272 kPa and the tensile strength increased from 44 to 272 kPa.

## 1. Introduction

Environmental consciousness has led to an increase of interest in the development of more sustainable and environmentally friendly materials from renewable resources which could replace the traditional ones. For this reason, green building strategies can be extremely efficient in terms of fossil fuel savings and greenhouse gas reduction [1]. Currently, wood waste (WW) causes an important economic and environmental issue. Recently, the authors of References [2,3] have reported that the European Union generates more than 50 million m^3^ of WW each year, and according to this data, the future potential of WW recycling is quite low, mainly lacking sustainable reuse or recycling applications, e.g., in the construction industry [4,5]. However, WW is highly biodegradable and easily attacked by microorganisms and insects, which shortens its service life. In order to extend its service life and increase its durability, wood is modified by physical or chemical methods. Thus polluting WW with various inorganic and organic contaminants. Such contaminants are a real issue in waste management which can impact not only on health and the environment, but also on their further reuse in specific applications [6].

Considering the growing interest of natural fillers to be used for the production of composites with various polymeric matrices, WW is an alternative source which can be immobilised in foamed polymers. Among the possible fillers, the forestry and/or agricultural waste stands out because of low cost and huge accessibility, as well as for the presence of free hydroxyl (–OH) groups susceptible to link to isocyanate (–NCO) groups, which confers a sufficient compatibility with the polyurethane (PUR). Therefore, the current studies focussed on the possibility of finding a more sustainable approach in order to reuse WW-based fillers in PUR biocomposite foams for building insulation. It is well known that one of the most adaptable polymers produced as solid or foamed products are PUR. PUR foams are an ideal solution for lightweight, low-energy buildings and constitute 23% of all PUR foams’ use [7].

Incorporation of natural fillers in PUR matrices is a well-known practice. For example, authors in Reference [8] have tested untreated and NaOH-treated wood fibres in flexible polyurethane foams. They found out that chemical treatment changes the ratio between open and closed pores, thus improving the sound resistance ability of the obtained products. Additionally, the authors of Reference [9] studied pineapple leaves-based cellulose fibres which were further chemically treated to remove hemicellulose, lignin and other soluble materials. It was observed that the chemical treatment did not enhance the mechanical performance and on the contrary, it reduced the cross-link ability between the filler and polymer matrix. The authors of Reference [10] showed that the silanisation of walnut shells produced PUR biocomposite foams with improved physical and mechanical properties such as compressive and flexural strength, as well as thermal conductivity. Alkaline treatment was shown to be successful for hemp fibres, an 8% concentration showed an improvement in interfacial adhesion between the hemp fibre and the PUR matrix [11]. Even though improvements might be observed for chemically treated natural fillers, similar improvements might be obtained for untreated fillers as well. Authors in References [12,13] demonstrated that the addition of hemp fibres improved the tensile and flexural behaviour of PUR biocomposite foams. Moreover, pine bark showed a great potential to be used in PUR foams where greater ultraviolet resistance is required, while wood chips produced brighter foams with a better photodegradation effect compared to darker ones [14].

All these studies show that WW-based fillers or aggregates have a great potential to be used in PUR biocomposite foams. Therefore, the aim of this study is to test five different WW-based fillers, i.e., uncleaned and cleaned pinewood sawdust (uWS and WS, respectively), pinewood bark (WB), hemp shives from Lithuania (HS-LT) and hemp shives from France (HS-FR), and select the one with the best potential for the incorporation in loose-fill PUR biocomposite foams with different binder/filler ratios. Additionally, the obtained products will be subjected to testing of the most common performance characteristics.

## 2. Experimental

### 2.1. Materials and Methods for the Selection of WW-Based Filler

The following WW materials were selected to be tested as possible fillers for the preparation of loose-fill biocomposites: uncleaned and cleaned pinewood sawdust (uWS and WS, respectively), as well as pinewood bark (WB), were received from the Dzukija region forest in Lithuania (Alytus). Hemp shives (HS-LT) and (HS-FR) were delivered from Lithuanian (Rokiskis) and French (Bar sur Aube) farmers, respectively. The main physical characteristics of all the WW-based fillers are presented in Table 1.

The bulk density of WW-based fillers was determined according to the standard methodology as presented in Reference [15]. The empty dish was weighed and filled to overflowing with the respective filler using a spoon. In order to ensure an even surface, the surplus was carefully removed, then the dish was weighed, and the bulk density of each filler was calculated. Moisture content was determined, according to the method outlined in Reference [16], at 70 ± 2 °C temperature until the changes between three consecutive weightings, made 24 h apart, did not change by 0.1% of the total mass.

Thermal conductivity of WW-based fillers was determined for three 300 × 300 × 100 mm-sized specimens using a heat flow meter apparatus FOX 304 (LaserComp, New Castle, DE, USA), which has the measurement limits from 0.01 to 0.50 W/m·K and a measuring accuracy of ~1%. The difference between the measuring plates was 20 °C while the average test temperature was 10 °C. The conditioning procedure was taken from the harmonised wood wool standard [17]. Before the test, the specimens were stored at 23 ± 2 °C temperature and 50% ± 5% relative air humidity conditions until the changes were no more than 0.5%.

The microstructural analysis of the WW-based fillers was conducted using a JEOL JSM-7600 F (JEOL, Tokyo, Japan) scanning electron microscope having a resolution of 1.5 nm and magnification of up to 1 million times. Before the test, the prepared specimens were sputter-coated with a thin layer of gold under a vacuum environment.

Long-term water absorption by partial immersion was conducted based on method 1, as outlined in Reference [18], for 200 × 200 × 100 mm-sized porous cages filled with WW-based fillers. The total duration of the test was 28 days. Measurements for each specimen were taken each hour for 7 h, each day for 10 days, and every 4 days from 10 to 28 days. Water vapour permeability of the WW-based fillers was carried out on specimens with the dimensions of 100 × 100 × 50 mm. The assembly of the specimen and a dish with the salt was exposed to the surrounding environmental temperature of 23 ± 1 °C and relative humidity of 50% ± 3%. The relative humidity in the sealed assembly that was formed using sodium dichromate solution, in accordance with Reference [19], was 0%.

### 2.2. Materials, Preparation and Methods for PUR Biocomposite Foams

Rapeseed polyol, BioPolyol RD (SIA PolyLabs, Riga, Latvia), was used as one of the main components for the polyurethane binder (PURb). It has a hydroxyl value of 350 mg KOH/g and a water content <0.2%. Lupranat M20S (31.5% NCO) (BASF, Berlin, Germany), a polymeric 4,4-diphenylmethane diisocyanate, was used as the second component for the synthesis of PURb. As a surfactant, a polyether-modified dimethyl-polysiloxane surfactant, Struksilon 8006 (Brenntag, Kędzierzyn-Koźle, Poland), was selected for the PUR biocomposite foam compositions. Polycat 9 (Air Products and Chemical, Inc., Allentown, PA, USA) was used as a catalyst to control a reaction start and end time of the mixture. For the preparation of biocomposites with a WS filler and PURb, different ratios, i.e., 0.4, 0.55, 0.70 and 0.85, were chosen.

The compositions of the prepared PUR biocomposite foams are presented in Table 2. Firstly, the rapeseed polyol, catalyst and surfactant were stirred together for 1 min at 1800 rpm. Further, the obtained mixture was mixed with the appropriate weight of isocyanate and stirred for 10 s to obtain the PURb. Then, the PURb was poured onto a weighed amount of WS filler and thoroughly mixed for 1 min at 1800 rpm. The whole mixture was freely poured into 300 × 300 × 50 mm moulds and kept for another 24 h before being demoulded and cut into the specified sizes.

The apparent density of the PUR biocomposite foams was measured according to the requirements mentioned in Reference [20]. Three specimens with a size of 100 × 100 × 50 mm were tested, and the average value was calculated. Before the apparent density determination, specimens were conditioned ≥6 h at 23 ± 5 °C.

Compressive strength was determined for 100 × 100 × 50 mm-sized specimens according to Reference [21] using a computerised testing machine, Hounsfield H10KS (Hounsfield, Surrey, UK), with a maximum loading force of 10 kN, a loading accuracy ± 0.5% and a loading speed accuracy ± 0.05%. Before the test, the specimens were conditioned ≥6 h at 23 ± 5 °C. Then, the specimen was aligned onto the bottom support of the machine and loaded with an initial loading of 250 ± 10 Pa. During the test, loading speed was 0.1 d ± 25% mm/min. Tensile strength testing was conducted on 50 × 50 × 50 mm-sized specimens according to Reference [22], perpendicular to the foaming direction of PUR biocomposite foams. For the test, the same computerised universal machine was used as for the compressive strength determination.

Thermal conductivity of WS-filled PUR biocomposite foams was determined based on Reference [23] for five 300 × 300 × 100 mm-sized specimens of each composition, using a heat flow meter apparatus FOX 304 (LaserComp, New Castle, DE, USA), which has the measurement limits from 0.01 to 0.50 W/m·K and a measuring accuracy of ~1%. The temperature difference between the measuring plates was 20 °C while the average test temperature was 10 °C. The conditioning procedure was the same as for the WW-based fillers.

The microstructural analysis of the PUR biocomposite foams with varying PURb/WS ratios was conducted with a JEOL JSM-7600 F (JEOL, Tokyo, Japan) scanning electron microscope having a resolution of 1.5 nm and magnification of up to 1 million times. Before the test, the prepared specimens were sputter-coated with a thin layer of gold under a vacuum environment. In order to evaluate the interface between the WS filler and PUR biocomposite foams, optical images were taken with an optical microscope Smart 5M PPRO (Delta Optical, Gdańsk, Poland).

The number of closed cells was determined in accordance with method 2, as outlined in Reference [24], for three 30 × 30 × 100 mm-sized specimens of each composition. Average cell size of PUR biocomposite foams was measured from scanning electron microscope images using ImageJ software (1.52a, National Institute of Health, Bethesda, MD, USA).

Short-term water absorption by partial immersion was conducted based on Reference [25], method 1, for 200 × 200 × 100 mm-sized WS-filled PUR biocomposite foam specimens. The total duration of the test was 24 h. Water vapour permeability was carried out according to the same procedure as for WW-based fillers.

The dimensional stability test was conducted according to Reference [26] on 200 × 200 × 50 mm-sized specimens at −20 °C temperature, 70 °C temperature and 90% relative humidity conditions for 48 h. Before the test, the specimens were conditioned at 23 ± 2 °C temperature and 50% ± 2% relative humidity for 14 days until the changes in linear dimensions were < 0.1%.

## 3. Results and Discussion

### 3.1. Selection and Characterisation of the WW-Based Filler

**Figure 1:**
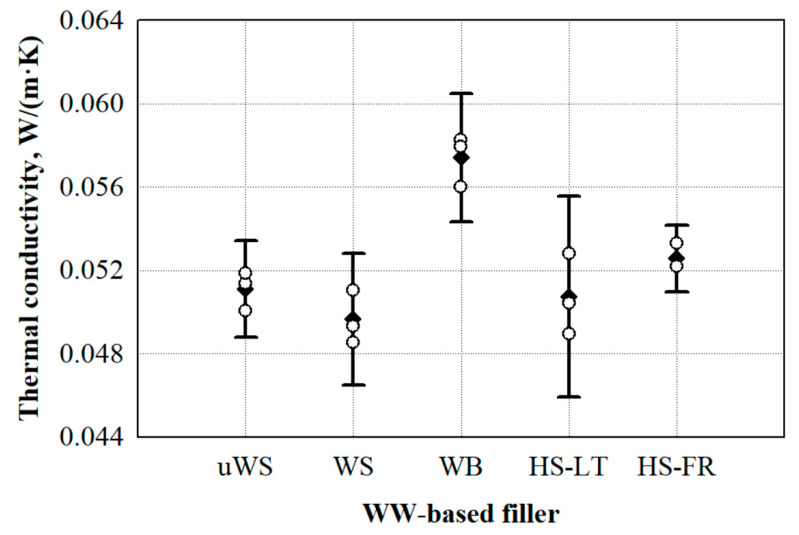
Thermal conductivity variation in regards of the type of WW-based filler: uWS—uncleaned sawdust, WS—cleaned sawdust, WB—wood bark, HS-LT—Lithuanian hemp shivs, HS‑FR—French hemp shivs. ο—single values from thermal conductivity measurements; ●—average value from thermal conductivity measurements.

Whereas the selection of the WW-based filler is for the further preparation of loose-fill PUR biocomposite foams which are processed without any additional loading, the average values of thermal conductivity for each filler are presented based on their measured bulk density (Figure 1).

Taking into consideration the scattering of the results, it can be seen from Figure 1 that the thermal conductivity values of most of the WW-based fillers are in the range of 0.046–0.061 W/m·K, while the WB filler is characterised by the highest thermal conductivity value. Additionally, an almost ~10% difference between HS-LT and HS-FR can be observed. This might be attributed to the size of the hemp particles and the bulk density. Compared to HS-FR, the HS-LT filler has 30% more 2.5–5 mm-sized particles, and a previous study in Reference [27] confirms that the optimal thermal insulating properties of HS fillers are obtained when the HS particles are further shredded, or their size ranges from 2.5 to 5 mm. This is explained by the fact that, compared to larger particles, smaller voids and air gaps are formed between smaller HS particles through which a lower heat exchange occurs due to heat transfer through gas. Additionally, it is clearly seen from Table 1 that HS-FR has 19% greater bulk density than that for HS-LT. The increase in thermal conductivity value based on bulk density is in good agreement with a study conducted in Reference [28].

**Figure 2:**
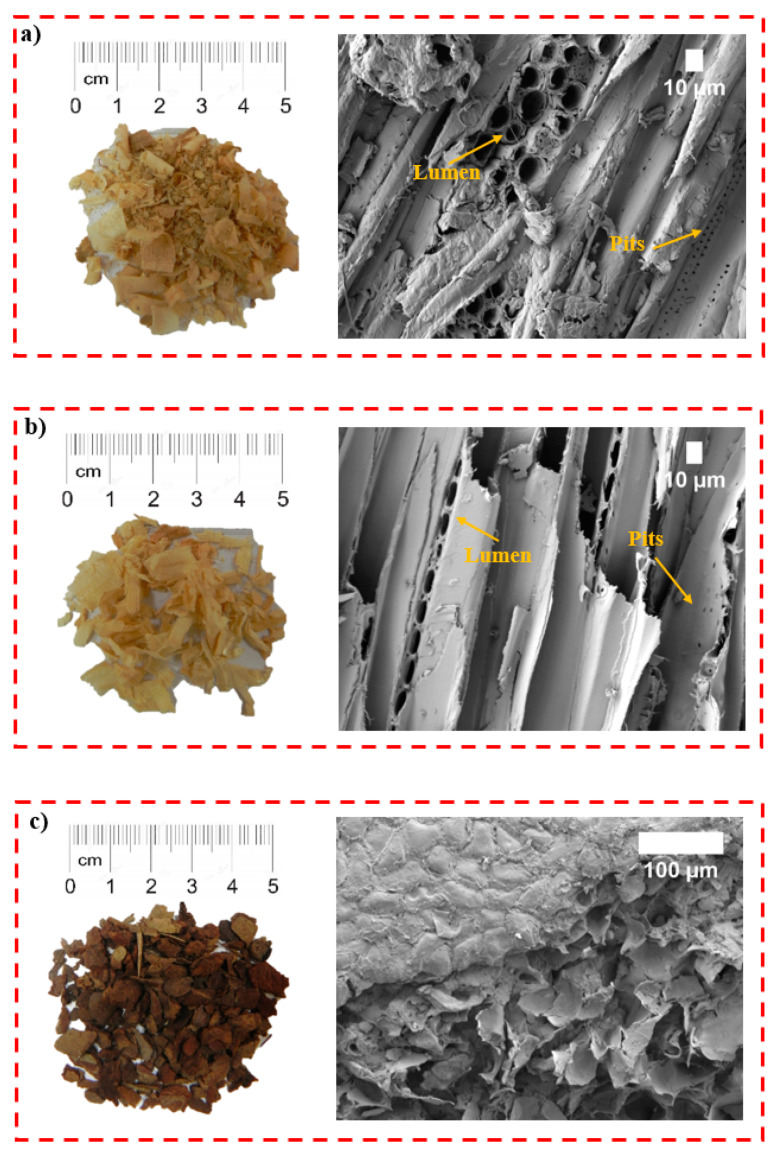

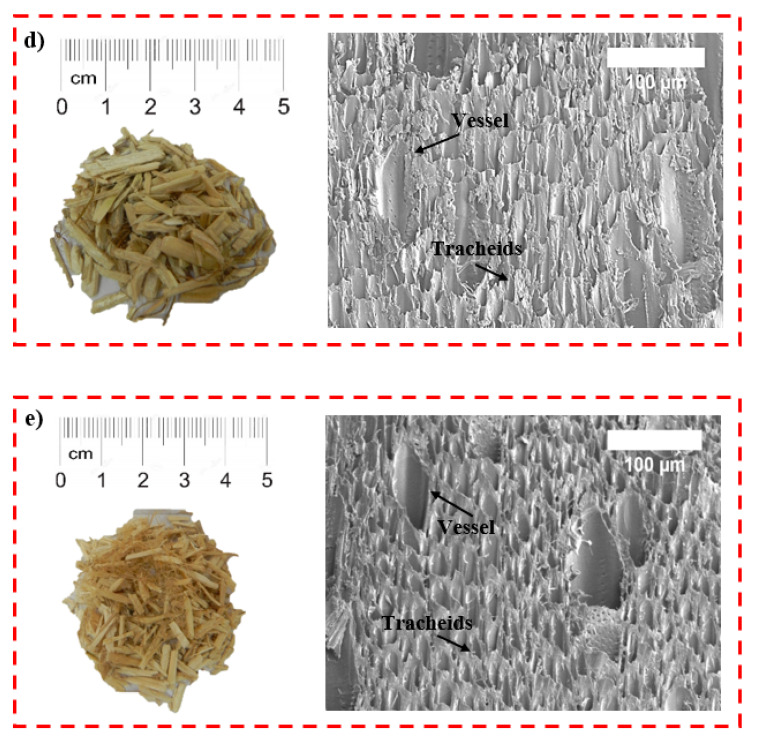
General views and scanning electron microscope (SEM) images of WW-based fillers: (**a**) uWS (magnification ×500), (**b**) WS (magnification ×500), (**c**) WB (magnification ×250), (**d**) HS-LT (magnification ×500) and (**e**) HS-FR (magnification ×500).

It can be clearly seen that the lowest thermal conductivity was determined for the WS filler. Compared to uWS, WB, HS-LT and HS-FR, the thermal conductivity of WS is lower by 3.0%, 16%, 2.2% and 6.0%, respectively. The differences in these values may be explained by the filler’s structure. Therefore, Figure 2 presents the microstructures of the WW-based fillers.

It can be seen from Figure 2a,b that uWS and WS particles show the traditional structure of wood with hollow lumen spaces which are visible in the cross-section, and numerous pits across longitudinal areas.

The pore sizes of uWS and WS are of 10–20 μm with approximately ~2 μm-sized pits located in the tracheid’s walls. Additionally, the surfaces of the uWS filler particles are covered with fine particles, of which 24.3% are of < 0.315 mm. Accordingly, this increases the average thermal conductivity by 2.2% compared to the WS filler (the content of < 0.315 mm particles is 0.89%) due to the increased heat transfer via solid components. However, the highest thermal conductivity value is observed for the WB filler. As can be seen from Figure 2c, WB contains mainly two types of pores whose size varies from 20 to 100 μm, which is confirmed by the research in Reference [29]. Taking into consideration the amount of < 0.315 mm-sized particles in the WB filler, i.e., ~8%, this might be the reason for the increased heat transfer through the solid parts and a higher thermal conductivity value compared to the other WW-based fillers.

**Figure 3:**
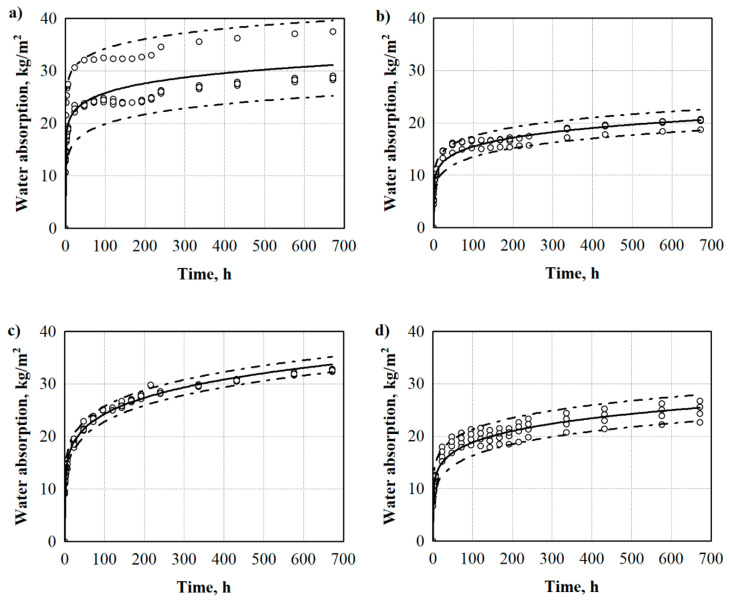

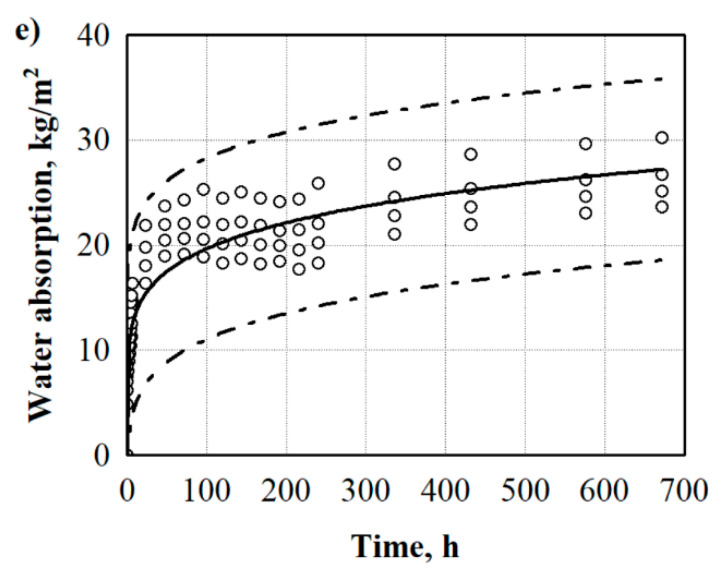
Long-term water absorption by partial immersion of WW-based fillers: (**a**) uWS, (**b**) WS, (**c**) WB, (**d**) HS-LT and (**e**) HS-FR. uWS—uncleaned sawdust, WS—cleaned sawdust, WB—wood bark, HS-LT—Lithuanian hemp shivs, HS-FR—French hemp shivs, ο—single values from water absorption measurements.

It is known that the WW-based fillers have many pores in their structure, and they include a large number of hydrophilic groups. Therefore, hygroscopicity of the WW-based fillers occurs in wet environmental conditions and this can lead to deformation, cracking and other defects of products made from them [30], thus making it important to know their behaviour under the impact of moisture. The water absorption ratio of different WW-based fillers is shown in Figure 3. As can be seen, the water absorption rate was so fast that it almost reached its maximum value in 24 h, the values being 22.4 kg/m^2^ for uWS, 12.5 kg/m^2^ for WS, 19.0 kg/m^2^ for WB, 15.0 kg/m^2^ for HS-LT and 15.4 kg/m^2^ for HS-FR. Between 14 and 672 h, the water absorption rate increased slowly, and thereafter it remained nearly constant for all the WW-based fillers. A similar pattern for water absorption increase was observed in Reference [31] for pine wood under no, and various, heat treatments. Strangely, even though uWS and WS fillers are almost the same, the average values of long-term water absorption by partial immersion after 24 h of soaking in water differ by almost 2 times. It is noticed from a study conducted in Reference [32] that in the comparison of coarse and fine fillers, fine WW-based particles attract and absorb more water than coarse ones. Therefore, the greater water absorption of uWS, which has the highest content of fine < 0.315 mm-sized particles (Table 1), can be seen. This is primarily because of increased surface area and greater effective absorption capacity of the fine particles as compared to coarse particles. In principle, uWS has more fine particles (>20%) than any other filler involved in this study. The same can be attributed to the WB filler which has the second highest amount of fine particles, i.e., >8%, therefore, its water absorption value after 24 h is ~15% lower compared to uWS. Moreover, it was found out by the authors of Reference [33] that a higher amount of lignin reduces the water retention ability of WW-based fillers as WB particles have ~37–40% lignin, while, e.g., WS has ~25% [34]. The third type of WW-based fillers analysed are HS which, depending on their country of origin, show quite similar tendencies over the test time and an almost identical average water absorption value. However, water absorption results of the HS-FR filler are characterised by a wide scattering. This can be explained by the fact that HS-FR filler consists of hemp shives and accumulated fibres, which determined the uneven test results.

In order to evaluate the scattering of the results for all WW-based fillers, mathematical-statistical calculations and interpretations of the results were implemented. Statistical data of long-term water absorption by partial immersion results are presented in Table 3.

Research shows that the obtained results of water absorption can be approximated by the regression equation. Table 3 presents the constant coefficients *b*_0_ and *b*_1_ the average standard deviation *S_r_*, and the determination coefficient $R_{t\cdot W_{lp}}^{2}$. The obtained data show that the determination coefficients for WW-based fillers are 0.779, 0.962, 0.993, 0.961 and 0.751, for uWS, WS, WB, HS-LT and HS-FR, respectively. They show that the suggested regression model is suitable to describe the water absorption values of WW-based fillers. Additionally, determination of the coefficients for uWS and HS-FR water absorption shows that a high amount of fine particles and fibres in the fillers causes a higher scattering of the obtained results.

**Figure 4:**
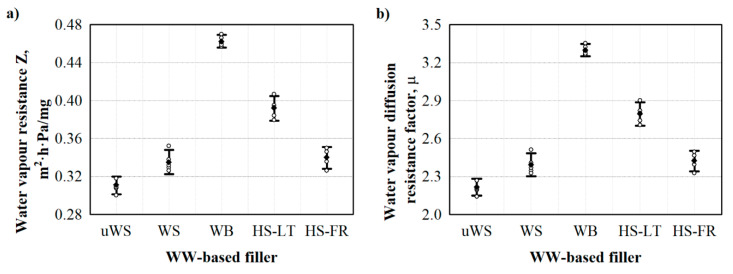
Water vapour permeability parameters of WW-based fillers: (**a**) water vapour resistance and (**b**) water vapour diffusion resistance factor. uWS—uncleaned sawdust, WS—cleaned sawdust, WB—wood bark, HS-LT—Lithuanian hemp shivs, HS-FR—French hemp shivs, ο—single values from water vapour parameters measurements, ●—average value from water vapour parameters measurements.

As the authors of Reference [35] stated, bark is less hygroscopic than wood, and the water and humidity sorption/desorption behaviour of WW-based fillers plays an essential role in the heating energy consumption of buildings. Therefore, water vapour permeability tests were conducted, and the results of the water vapour resistance and water vapour diffusion resistance factor are presented in Figure 4. It can be clearly seen that the WB filler has the highest water vapour resistance and water vapour diffusion resistance factor values, which are approximately 1.5 times higher compared to the uWS filler. This can be attributed to the highest bulk density amongst all the fillers. Similar observations were reported by the authors of Reference [36], who investigated the water vapour diffusion resistance factor values of several commercially available wood-based products, with different densities and thicknesses, and found that the water vapour diffusion resistance factor of the wood-based products increased with increasing density. Additionally, the WB filler has the lowest possible porosity (~60 vol.% [37]) compared to WS (~78 vol.% [38]) and HS (~77 vol.% [39]) fillers, which might be another key factor influencing the increased water vapour resistance.

Furthermore, it is possible to observe that the water vapour resistance properties obtained for the WW-based fillers are in the range of 2.2–3.3, which are in a good agreement with the values of vegetable fibres, such as wood (3–10), hemp, flax and corn (1–3), as presented in Reference [40].

### 3.2. Apparent Density, Thermal Conductivity and Microstructure of WS-Filled PUR Biocomposite Foams

**Figure 5:**
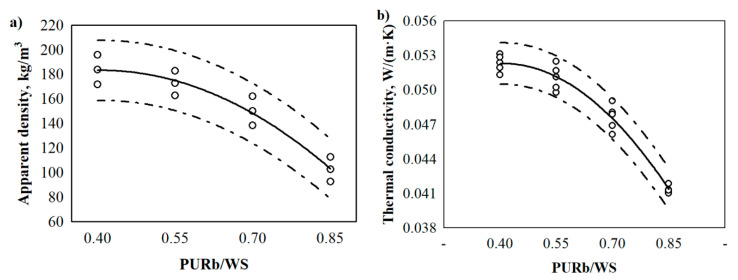
The changes of physical properties of PUR biocomposite foams with different PURb/WS ratios: (**a**) apparent density, (**b**) thermal conductivity. WS—cleaned sawdust, ο—single values from respective property measurements.

Application of the renewable raw materials, especially wood-based ones, in the synthesis of biocomposite polyurethane foams can significantly change their properties [41,42]. Regarding the application of a current PURb/WS biocomposite foam as a loose-fill thermal insulation for building envelopes, the determination of performance properties such as apparent density, thermal conductivity, compressive and tensile strengths, water absorption, water vapour permeability, dimensional stability and structural changes, are of great importance. Therefore, the changes in apparent density and mechanical behaviour of biocomposites with 0.40, 0.55, 0.70 and 0.85 of PURb/WS ratio are illustrated in Figure 5.

It is seen that an increase in the PURb/WS ratio reduces the apparent density of PUR biocomposites. Compared to 0.40 PURb/WS, the apparent density of 0.85 PURb/WS is reduced by approximately 44%. The highest apparent density of PUR biocomposite foams at the lowest PURb/WS ratio is explained by the fact that no additional blowing agent was used for the preparation of the samples. Additionally, a lower ratio of PURb/WS indicates a lower amount of PURb, which hinders the foaming process of PUR biocomposite foams. The results of previous studies of PUR biocomposite foams modified with other fillers show that an incorporation of, e.g., industrial potato protein [43], solid waste from leather industry [44], or algal cellulose [45], reduce the reactivity of the overall system, thus increasing the apparent density of the resultant PUR products. However, a further increase in PURb/WS ratio leads to a more effective blowing efficiency due to an increase in the PURb amount, which produces a higher reaction ability and CO_2_ emission as the –OH groups from polyol and –NCO groups from isocyanate react with each other.

The thermal conductivity of biocomposites with different PURb/WS ratios is mainly affected by the apparent density, porosity, pore size distribution, moisture content and phase composition [46]. However, the same raw materials and conditions were adopted in the tests, and therefore, the impact of moisture content and phase composition is not prominent. Moreover, in previous studies [47,48], pores were reported to be one of the main influencing factors on the value of thermal conductivity. Therefore, the average values of closed cell content for PUR biocomposite foams with different PURb/WS ratios are presented in Table 4, and thermal conductivity values in Figure 5b.

**Figure 6:**
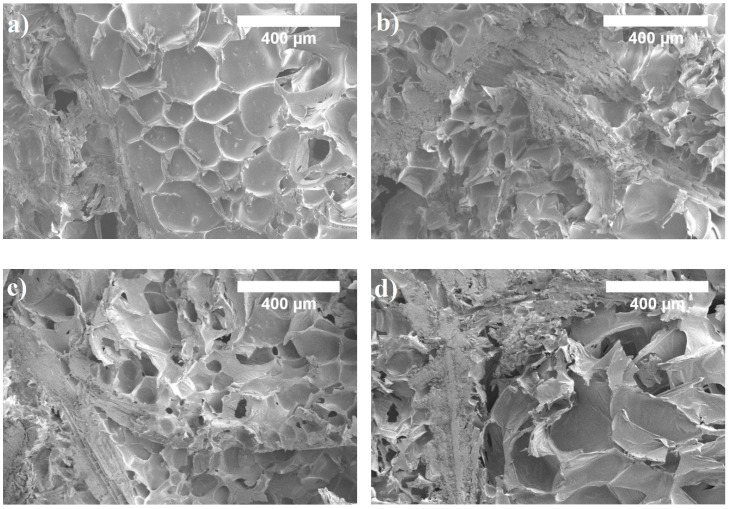
Microstructure of PUR biocomposite foams with different PURb/WS ratios: (**a**) 0.40 PURb/WS, (**b**) 0.55 PURb/WS, (**c**) 0.70 PURb/WS and (**d**) 0.85 PURb/WS (magnification ×150).

It shows that the lowest and the highest ratios of PURb/WS determined the PUR biocomposite foams with the lowest volumetric content of closed cells, i.e., approximately 50 vol.%, while 0.70 PURb/WS biocomposite foam reached almost 70 vol.%. It can be seen from Figure 6a,d that the interface interaction between the porous polyurethane matrix and WS particles is not even, it is full of voids and flaws which increases the percentage of open pores and reduces the percentage of closed ones.

**Figure 7:**
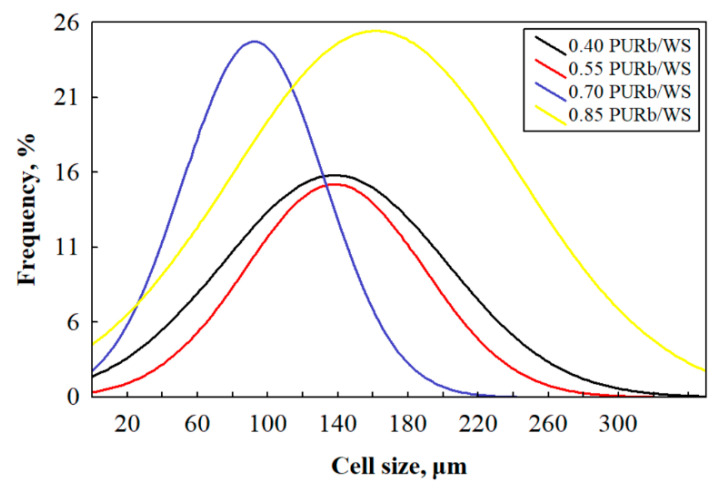
Cell size distribution of PUR biocomposite foams with different PURb/WS ratios.

In the case of 0.40 PURb/WS, the excessive amount of WS particles, or the insufficient amount of PURb, disrupts the foaming process and results in the formation of a more defective microstructure, which determines a higher average value of thermal conductivity, which is 0.0523 W/m·K. The observations made are in good agreement with previous studies of polyurethane foams with various loadings of natural fillers [10,49,50]. However, a 0.85 PURb/WS ratio allows a higher blowing efficiency of PUR biocomposite foams due to the increased capability of the PURb mixture to react with the moisture present in WS particles as the PURb amount increases. Consequently, some of the cell walls cannot withstand the pressure during excessive blowing, thus reducing the overall closed cell content and the thermal conductivity by approximately 19% and 13%, as compared to 0.70 PURb/WS. The observation made is also proven by the average cell size (Table 4) and cell size distribution measurements in Figure 7. It is calculated that the use of a 0.85 PURb/WS ratio determines by, on average, a 74% higher cell size compared to a 0.70 PURb/WS ratio and shifts cell size distribution curve towards higher sizes.

With the increasing ratio of PURb/WS, the overall cell structure becomes less uniform and the number of broken cells is increased. In the case of 0.40–0.70 PURb/WS ratios, the cellular structure is quite well preserved, and the reduction in cell size is visible (Table 4 and Figure 7). This indicates that the application of up to 0.70 PURb/WS enhances the formation of smaller cells due to the existence of small particles in the WS filler. It is known that the addition of filler may change the nucleation mode from homogeneous to heterogeneous, and reduce the nucleation energy, thus promoting the formation of a higher number of smaller cells [51,52]. This phenomenon can be observed for the PUR biocomposite foams with a 0.70 PURb/WS ratio (Figure 6c). When the ratio of PURb/WS exceeds 0.70, damaged cells become visible (Figure 6d).

In order to evaluate the impact of the PURb/WS ratio on the apparent density and thermal conductivity of the obtained PUR biocomposite foams, statistical-mathematical analysis was implemented and data for regression Equations (1) and (2) is presented in Table 5.

(1) $$\rho_{r_{PURb/WS}}=b_{0}+b_{1}\cdot r_{PURb/WS}+b_{2}\cdot r_{PURb/WS}^{2}$$

(2) $$\lambda_{r_{PURb/WS}}=b_{0}+b_{1}\cdot r_{PURb/WS}+b_{2}\cdot r_{PURb/WS}^{2}$$

where: $\rho_{r_{PURb/WS}}$—the average apparent density of PUR biocomposite foams, $\lambda_{r_{PURb/WS}}$—the average thermal conductivity of PUR biocomposite foams, $r_{PURb/WS}$—PURb/WS ratio in PUR biocomposite foams and *b*_0_, *b*_1_ and *b*_2_—constant coefficients of regression equations (Table 5).

The statistical analysis shows that the results of apparent density and thermal conductivity can be approximated by the regression equation. Table 5 presents the constant coefficients *b*_0_, *b*_1_ and *b*_2_, the average standard deviation *S_r_*, and the determination coefficient $R_{r\cdot PURb/WS}^{2}$. The obtained data show that the determination coefficients for apparent density and thermal conductivity are 0.959 and 0.966, respectively. They show that the suggested regression models are suitable for the description of PUR biocomposite foam’s apparent density and thermal conductivity.

### 3.3. Strength Properties and Interfacial Adhesion of WS-Filled PUR Biocomposite Foams

**Figure 8:**
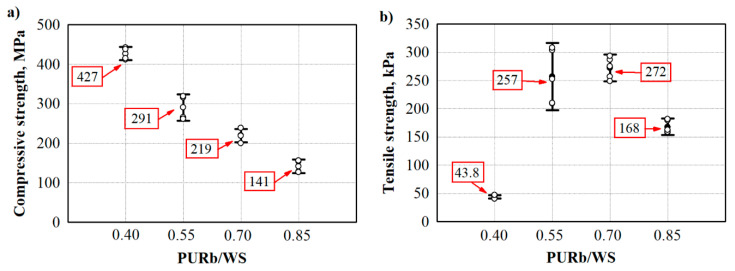
Strength properties of PUR biocomposite foams with different PURb/WS ratios: (**a**) compressive strength and (**b**) tensile strength. WS—cleaned sawdust, ο—single values from respective mechanical property measurements, ●—average value from respective mechanical property measurements.

The mechanical performance of cellular materials is closely dependent on their apparent density as well as their structural parameters, such as closed cell content and cells’ shape and sizes [53]. Therefore, it is of great importance to know how the PURb/WS ratio alters the overall mechanical properties of PUR biocomposite foams. Hence, the average numerical values of compressive and tensile strengths are presented in Figure 8. It can be seen that the compressive strength of PUR biocomposite foams with different PURb/WS ratios reduces with the reduction in apparent density. The highest value of compressive strength is reached for PUR biocomposite foams at a 0.40 PURb/WS ratio. The increase in PURb triggers a decrease in the compressive strength of the PUR biocomposite foams by ~32%, ~49% and ~70% respectively, for 0.55 PURb/WS, 0.70 PURb/WS and 0.85 PURb/WS. This happens due to the increased blowing efficiency under the reaction between moisture in the WS particles and isocyanate as one of the main components in the PURb mixture.

Although enhancement of the PUR biocomposite foams’ compressive strength was not observed, it is still important to address the required standard for insulation materials (≥120 kPa) [9]. This shows that the PUR biocomposite foams may be comparable to commercially available ones only at 0.40–0.55 PURb/WS ratios (≥250 kPa) [54].

Despite the reduction in compressive strength with the addition of PURb, the tensile strength (Figure 8b) shows a quite different tendency. It can be seen that the lowest tensile strength is obtained at 0.40 PURb/WS while 0.70 PURb/WS gives the highest value of the parameter, i.e., with the addition of PURb, the tensile strength increased by 621% compared to 0.40 PURb/WS, giving an average value of 272 kPa, indicating an improved interfacial adhesion and improved stress transfer during the stretching process between WS particles and PURb. Similar observations were reported in Reference [55], where microcellular polyurethane with sisal fibres were investigated, and in Reference [56], where research was conducted on waterborne polyurethane with cellulose reinforcement.

However, further additions of PURb negatively affects the tensile strength of PUR biocomposite foams at 0.85 PURb/WS. This effect can be attributed to the reduced density and, presumably, the thickness of the cell walls, which determines the overall tensile behaviour of PUR biocomposite foams.

**Figure 9:**
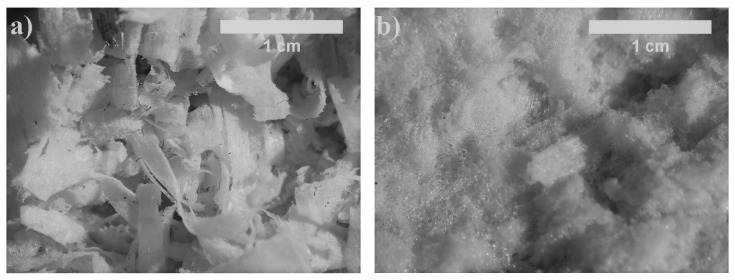
Surfaces of PUR biocomposite foams after tensile test: (**a**) 0.40 PURb/WS and (**b**) 0.70 PURb/WS.

Moreover, as presented in Figure 9, WS particles are not completely built in the PUR matrix, some of them are loose and pulled out from the matrix at a 0.40 PURb/WS ratio. This indicates poor interfacial adhesion and insufficient wetting between the WS surface and the PU matrix at a 0.40 PURb/WS ratio, which in turn leads to voids and the formation of open pores in the foam structure (Table 4). This can be attributed to the lower amount of PURb used for the formation of PUR biocomposite foams. Furthermore, PUR biocomposite foams at a 0.70 PURb/WS ratio are characterised by a more regular structure with a lower content of open cells compared to PUR biocomposite foams at a 0.40 PURb/WS ratio. In this case, WS particles are sufficiently covered with the PU matrix, thus leading to a better interfacial interaction and greater tensile strength values.

### 3.4. Water and Water Vapour Resistance of WS-Filled PUR Biocomposite Foams

**Figure 10:**
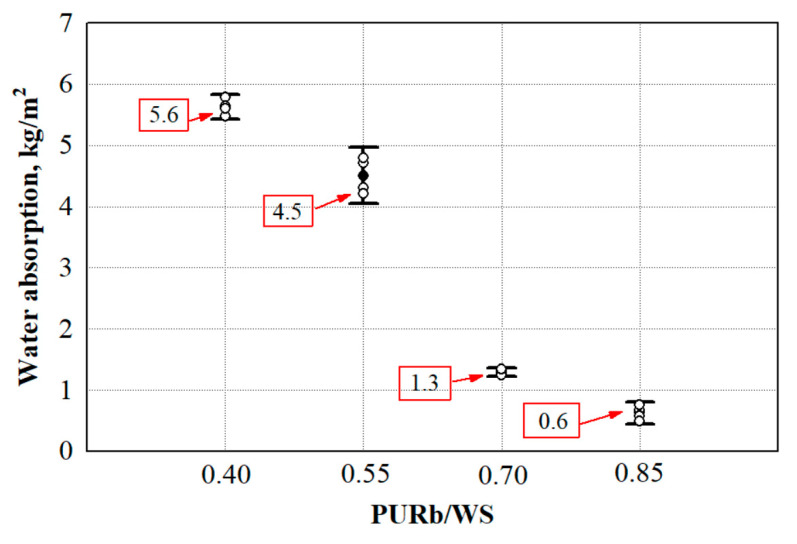
Short-term water absorption by partial immersion of PUR biocomposite foams. WS—cleaned sawdust, ο—single values from water absorption measurements, ●—average value from water absorption measurements.

Water absorption is another significant performance characteristic that has to be examined because it is highly important for the biodegradability of WW-based products [57]. Pure synthetic matrices are inert to water absorption, which is the main reason for their long period of degradation, while WW-based products are more attractive to microorganisms which is due to their capability to absorb water [58]. Therefore, the impact of water or water vapour on the obtained PUR biocomposite foams must be known, and the results are presented in Figure 10 and Table 6.

It is obvious that the increase in the amount of polymer matrix PURb reduces the total water absorption of PUR biocomposite foams. Compared to 0.40 PURb/WS, the parameter reduces by 20% for 0.55 PURb/WS, by 77% for 0.75 PURb/WS and by 90% for 0.85 PURb/WS ratios. The difference in water absorption and the change with the addition of PURb can be explained by the fact that at 0.85 PURb/WS, the WS particles are distributed in a higher volume, therefore, the hydrophobic matrix makes it harder for water to interact with WW-based filler, thus reducing the short-term water absorption by partial immersion values. This phenomenon has also been reported by other authors [59,60] who tested 24 h water immersion of pre-hydrolysed banana fibres-based polyurethane foam composites, and the contact angle of porous polyurethane filled with cellulose fibres from water hyacinth, respectively. Water vapour resistance is dependent on the thickness of the sample. Specimens with the edge size of 100 mm and thickness of 50 mm were used to determine the main parameters for the water vapour permeability test, water vapour resistance and water vapour diffusion resistance factor. It can be seen (Table 6) that a higher PURb amount produces a lower water vapour resistance and water vapour diffusion resistance factor.

This means that despite the closed cell structure, the use of WS filler increases the amount of water vapour transmitted through the material. The higher resistance to water vapour transport at 0.55 PURb/WS and 0.70 PURb/WS is due to a greater amount of PURb and a higher content of closed cells. It is obvious from Figure 4 that WS filler is sufficiently water vapour permeable, therefore, the obtained PUR biocomposite foams are characterised by a lower water vapour resistance and water vapour diffusion resistance factor compared to commercial ones. As a 0.40 PURb/WS ratio was considered insufficient for even wetting of the WS filler, it creates a path which can be easily accessed by water molecules penetrating through the specimens.

### 3.5. Dimensional Stability of WS-Filled PUR Biocomposite Foams

It known that the diffusion of gases accelerates with an increase in temperature. This is based on the basic polymer chemistry where gases in cells, influenced by a higher temperature, expand, thus exerting a pressure and changing the volume of the product. Moreover, the decrease in environmental temperature may impact the changes in linear dimensions. Therefore, it is of great importance to determine the influence of some standardised environmental conditions on PUR biocomposite foams with different PURb/WS ratios. The measured average percentage values of dimensional stability at 70 °C/90% and −20 °C are presented in Table 7.

The percentage linear changes in length, width and thickness after exposure at 70 °C/90% and −20 °C for up to 2 days for PUR biocomposite foams with different PURb/WS ratios are presented in Table 7. The dimensional stability of PUR biocomposite foams indicates that the increase in PURb/WS ratios (WS is constant and PURb increases according to the data presented in Table 2) results in negligible changes of dimensional stability of the foams. The relative changes of PUR biocomposite foams with all PURb/WS ratios in the directions of length and width, range from 0.5% to 1.4%, and in thickness, from 1.1% to 2.3%, when tested at 70 °C /90% conditions. According to the factory-made polyurethane foam harmonised product standard [61], the obtained biocomposite foams with 0.55–0.85 PURb/WS ratios fall into the fourth stability level and are characterised as extremely stable foams. However, the PUR biocomposite foams at a 0.40 PURb/WS ratio exceed the limits of the fourth level and fall into the third level. These slight changes might be attributed to the fact that WS particles in such PUR biocomposite foams are more exposed to a humid environment, which has a slightly negative effect on the final product. Additionally, according to the results presented in Table 7, PUR biocomposite foams when tested at −20 °C can be described as extremely stable and fall into the highest level indicated by the product standard. In each case, the dimensional stability of PUR biocomposite foams exposed to various environmental conditions are still considered to be stable and within commercially acceptable limits. Even though the test duration was only 2 days, similar conclusions have been made after 14 days of exposure at different conditions of PUR foam with pigment filler [62].

### 3.6. Comparison of the Obtained Results with the State-of-the-Art

After conducting a thorough literature review, Table 8 was concluded. It shows the types of WW-based fillers used for various polymers-based biocomposites and foams. The numerical values presented in various references are quite different. This can be attributed to the WW-based fillers amounts used for the preparation of products as well as the type and application of the product.

Higher values of compressive and tensile strengths are mostly obtained for PUR biocomposites used, e.g., for structural, decorative, furniture, etc., applications, while lower strength results are attributed to thermal insulation and sound-absorbing foams. The performance of biocomposite foams independently on the foamed matrix used is determined by the interface between fibre/aggregate and polymer binder. Additionally, the size of fillers/aggregates and the production technology with its parameters are of great importance as well.

Comparing the results of the thermal conductivity and mechanical performance of the current study and Table 8, it can be stated that the obtained WW-based PUR biocomposite foams are competitive to the ones already studied by other authors. However, there is a lack of information about short-term absorption by partial immersion of various polymers-based biocomposite foams. Therefore, it is hard to compare the obtained numerical values in Table 8 with the percentage values obtained in a current study.

## 4. Conclusions

In this study, various WW-based fillers, such as uWS, WS, WB, HS-LT and HS-FR, were investigated. The current research has shown that WS filler is characterised as the most suitable for building insulation properties with thermal conductivity—0.0496 W/m·K, short-term water absorption by partial immersion—12.5 kg/m^2^, water vapour resistance—0.34 m^2^·h·Pa/mg and water vapour diffusion resistance factor—2.4. Moreover, regression equations for the short-term water absorption of all WW-based fillers were also determined. As the most promising WW-based filler, WS was tested in polyurethane-based loose-fill material for building insulation application.

The ratios of 0.40 PURb/WS, 0.55 PURb/WS, 0.70 PURb/WS and 0.85 PURb/WS were used to form PUR biocomposite foams with the following properties: apparent density in the range of 103–184 kg/m^3^, thermal conductivity—0.0413–0.0523 W/m·K, compressive strength—142–427 kPa, tensile strength—44–272 kPa, short-term water absorption by partial immersion—0.63–5.56 kg/m^2^, water vapour resistance—0.96–1.7 m^2^·h·Pa/mg, water vapour diffusion resistance factor—15–26 and sufficient dimensional stability which conforms to the highest requirements for PUR foams. Additionally, the obtained results for apparent density and thermal conductivity were approximated by regression equations which show that the parameters are dependent on the PURb/WS ratio in PUR biocomposite foams by 96% and 97%, respectively.

It was found that the most efficient ratio is 0.70 PURb/WS, which gave stable products with the best properties for loose-fill building insulations, i.e., apparent density—150 kg/m^3^, thermal conductivity—0.0476 W/m·K, compressive strength—220 kPa, tensile strength—272 kPa, short-term water absorption by partial immersion—1.3 kg/m^2^, water vapour resistance—1.66 m^2^·h·Pa/mg and water vapour diffusion resistance factor—26. According to dimensional stability (DS) results, 0.70 PURb/WS ratio-based PUR biocomposite foams are highly stable in various environmental conditions and conform to DS (70, 90) level 4 for 70 °C/90% environmental conditions, and DS (−20) level 2 for −20 °C environmental conditions.

## Figures and Tables

**Table 1:** The main physical characteristics of WW-based fillers.

Characteristic	WW-Based Filler Type				
	uWS	WS	WB	HS-LT	HS-FR
Bulk density, kg/m^3^	93.2 ± 7.8	83.0 ± 6.8	210 ± 15	112 ± 9.3	133 ± 8.0
Granulometry, %					
≥10 mm	0.18	3.64	1.69	0.01	1.42
10–5 mm	13.8	43.4	13.2	1.59	3.23
5–2.5 mm	26.8	30.4	32.1	52.3	22.1
2.5–1.25 mm	6.92	14.3	9.81	42.1	61.3
1.25–0.63 mm	16.2	6.56	21.3	1.58	1.84
0.63–0.315 mm	11.8	0.81	13.7	1.78	7.35
<0.315 mm	24.3	0.89	8.20	0.64	2.76
Moisture content, %	10.4 ± 3.6	12.1 ± 1.9	15.8 ± 1.3	9.1 ± 0.33	10.5 ± 0.90

**Table 2:** Compositions of PUR biocomposite foams.

Material	PURb/WS			
	0.40	0.55	0.70	0.85
WS, g	800	800	800	800
PURb, g	320	440	560	680
Isocyanate	Index 100			

Note: the amount of additives was constant—1 g of Polycat 9 (blowing catalyst) and 3.5 g of Struksilon 8006 (surfactant).

**Table 3:** Statistical data of long-term water absorption by partial immersion results.

WW-Based Filler	No. of Samples	Equation * Coefficients		$R_{t\cdot W_{lp}}^{2}$	*S_r_*, kg/m^2^
		*b*_0_	*b*_1_		
uWS	92	16.40	0.0979	0.779	3.66
WS		7.831	0.1480	0.962	1.01
WB		10.98	0.1722	0.993	0.743
HS-LT		9.069	0.1582	0.961	1.28
HS-FR		8.988	0.1700	0.751	4.40

* Equation—$W_{lp}=b_{0}\cdot t^{b_{1}}$, where $W_{lp}$—long-term water absorption by partial immersion, *b*_0_, *b*_1_—coefficients which were calculated from the experimental data, *t*—duration, in hours. Note: Two-sided prediction confidence interval of the results is presented with 95% probability, Student’s criterion $t_{\alpha }=1.96$ when α=0.10 (dotted lines in Figure 3).

**Table 4:** Structural parameters of PUR biocomposite foams.

Parameter	PURb/WS			
	0.40	0.55	0.70	0.85
Closed cell content, vol.%	52 ± 4	62 ± 71	68 ± 5	55 ± 5
Cell size, μm	138 ± 12	138 ± 15	91.8 ± 10	161 ± 15

**Table 5:** Statistical data of apparent density and thermal conductivity results.

Parameter	No. of Samples	Equation Coefficients			$R_{r\cdot PURb/WS}^{2}$	*S_r_*	*t_α_*
		*b*_0_	*b*_1_	*b*_2_			
Apparent density, Equation (1)	12	115.0944	333.6444	−408.889	0.959	10.6	2.31
Thermal conductivity, Equation (2)	20	0.043089	0.045236	−0.055600	0.966	0.000858	2.10

**Table 6:** Water vapour permeability results of PUR biocomposite foams.

Parameter	PURb/WS			
	0.40	0.55	0.70	0.85
Water vapour resistance *Z*, m^2^·h·Pa/mg	0.958 ± 0.10	1.35 ± 0.15	1.66 ± 0.20	1.16 ± 0.12
Water vapour diffusion resistance factor *μ*, r. u.	15.2 ± 1	21.4 ± 1	26.3 ± 2	18.4 ± 2

Note: r. u.—relative units.

**Table 7:** Dimensional stability of PUR biocomposite foams with different PURb/WS ratios.

Parameter	PURb/WS			
	0.40	0.55	0.70	0.85
Dimensional stability at 70 °C and 90% conditions, %:				
length	1.4 ± 0.2	1.0 ± 0.3	0.8 ± 0.1	0.7 ± 0.1
width	1.1 ± 0.2	0.8 ± 0.1	0.7 ± 0.1	0.5 ± 0.1
thickness	2.3 ± 0.4	1.5 ± 0.3	1.1 ± 0.3	1.2 ± 0.2
Dimensional stability at −20 °C, %:				
length	0 ± 0	0 ± 0	0.5 ± 0.1	0.5 ± 0.2
width	0 ± 0	0 ± 0	0.5 ± 0.1	0.5 ± 0.3
thickness	1.1 ± 0.2	1.0 ± 0.3	1.0 ± 0.2	1.4 ± 0.3

**Table 8:** Properties of polymer-based biocomposite foams with WW-based fillers.

Filler Type	Amount of Filler, %, Amount of Binder, %	Parameter			
		Thermal Conductivity, W/(m·K)	Compressive Strength, kPa	Tensile Strength, kPa	Water Absorption, %
Pineapple leaf fibres (PLF) [9]	1–5% PLF, 100% PUR	-	220–340	-	-
Crystalline (CC) and amorphous cellulose (CA) [48]	10–20% CC and CA, 100% PUR	0.034–0.045	~400–800	-	-
Fir sawdust (FS) [1]	100% FS, 20–40% PUR	0.039–0.163	~70–100	-	-
Birch sawdust (BS) [1]	100% BS, 25–40% PUR	0.041–0.085	~90–100	-	
Rice waste (RW) [63]	5–20% RW, 100% PUR	-	-	150–250	-
Pine bark (PB) and peanut shell (PS) [64]	2–10% PB and PS, 100% PUR	-	100–140	-	-
Mable fibres (MF) [65]	13.3% MF, 100% PUR	-	28	92	-
Cork particles (CPs) [66]	100% CP, 10–50% PUF	0.090–0.120	-	600–1800	-
Kenaf fibres (KF) [67]	20% KF, 80% PLA	-	-	18,660	4.8
Cellulose powder (CP) [68]	33% CP, 100% PSC	-	-	1760–2250	350
Cellulose nanocrystals (CNC) [69]	2–8% CNC, 100% PUR	0.025–0.026	190–210	-	-
Date Pit (DP) [70]	10–40% DP, 100% PLA	0.079–0.068	70,000–80,000	-	0.2–5.5
Hemp fibres (HF) [12]	5–30% HF, 100% PUR	0.034–0.041	-	675–1413	29–65

Note: PUR—rigid polyurethane foam, PUF—flexible polyurethane foam, PLA—polylactic acid, PSC—potato starch.
